# Pilot Study on Mass Spectrometry–Based Analysis of the Proteome of CD34^+^CD123^+^ Progenitor Cells for the Identification of Potential Targets for Immunotherapy in Acute Myeloid Leukemia

**DOI:** 10.3390/proteomes6010011

**Published:** 2018-02-12

**Authors:** Johannes R. Schmidt, Elke Rücker-Braun, Katharina Heidrich, Malte von Bonin, Friedrich Stölzel, Christian Thiede, Jan M. Middeke, Gerhard Ehninger, Martin Bornhäuser, Johannes Schetelig, Kristin Schubert, Martin von Bergen, Falk Heidenreich

**Affiliations:** 1 Department of Molecular Systems Biology, Helmholtz-Centre for Environmental Research—UFZ, 04318 Leipzig, Germany; johannes.schmidt@ufz.de (J.R.S.); kristin.schubert@ufz.de (K.S.); martin.vonbergen@ufz.de (M.v.B.); 2 Department of Medicine I, University Hospital of Dresden, 01307 Dresden, Germany; Elke.Ruecker-Braun@uniklinikum-dresden.de (E.R.-B.); Katharina.Heidrich@uniklinikum-dresden.de (K.H.); Malte.Bonin@uniklinikum-dresden.de (M.v.B.); Friedrich.Stoelzel@uniklinikum-dresden.de (F.S.); Christian.Thiede@uniklinikum-dresden.de (C.T.); JanMoritz.Middeke@uniklinikum-dresden.de (J.M.M); Gerhard.Ehninger@uniklinikum-dresden.de (G.E.); Martin.Bornhaeuser@uniklinikum-dresden.de (M.B.); Johannes.Schetelig@uniklinikum-dresden.de (J.S.); 3 Center for Regenerative Therapies Dresden, TU Dresden, 01307 Dresden, Germany; 4 German Cancer Consortium (DKTK), 01307 Dresden, Germany; 5 German Cancer Research Center (DKFZ), 69120 Heidelberg, Germany; 6 National Center for Tumor Diseases, University Hospital Carl Gustav Carus, TU Dresden, 01307 Dresden, Germany; 7 DKMS German Bone Marrow Donor Center, Clinical Trials Unit, 01309 Dresden, Germany; 8 Institute of Biochemistry, Faculty of Life Sciences, Leipzig University, 04103 Leipzig, Germany

**Keywords:** hematopoietic stem/progenitor cells, acute myeloid leukemia, leukemic stem/progenitor cells, proteome

## Abstract

Targeting of leukemic stem cells with specific immunotherapy would be an ideal approach for the treatment of myeloid malignancies, but suitable epitopes are unknown. The comparative proteome-level characterization of hematopoietic stem and progenitor cells from healthy stem cell donors and patients with acute myeloid leukemia has the potential to reveal differentially expressed proteins which can be used as surface-markers or as proxies for affected molecular pathways. We employed mass spectrometry methods to analyze the proteome of the cytosolic and the membrane fraction of CD34 and CD123 co-expressing FACS-sorted leukemic progenitors from five patients with acute myeloid leukemia. As a reference, CD34^+^CD123^+^ normal hematopoietic progenitor cells from five healthy, granulocyte-colony stimulating factor (G-CSF) mobilized stem cell donors were analyzed. In this Tandem Mass Tag (TMT) 10-plex labelling–based approach, 2070 proteins were identified with 171 proteins differentially abundant in one or both cellular compartments. This proof-of-principle-study demonstrates the potential of mass spectrometry to detect differentially expressed proteins in two compartment fractions of the entire proteome of leukemic stem cells, compared to their non-malignant counterparts. This may contribute to future immunotherapeutic target discoveries and individualized AML patient characterization.

## 1. Introduction

During the past 15 years, mass spectrometry has undergone significant technological improvements allowing the rapid identification of large numbers of proteins today. New strategies of pre-fractionating the cell lysate help to reduce the complexity of the protein sample, thus enhancing coverage. This is accompanied with the development of novel protein and peptide labelling strategies which allow the semi-quantitative proteomic analysis of up to ten samples in one liquid chromatography mass spectrometry (LC-MS) run. The application of these state-of-the-art mass spectrometry-based methods may help to identify Acute Myeloid Leukemia (AML) stem cell biomarkers. A prerequisite for the use of an identified protein as an immunotherapeutic target molecule is its leukemia-specific expression, or at least the significant over-expression in leukemic cells. In the search for leukemia specific proteins, by applying two-dimensional electrophoresis combined with mass spectrometry methods, Ota et al. identified a limited average total number of 168 proteins in 13 samples of CD133^+^ hematopoietic stem cell-like cells from patients with various hematologic malignancies, including five AML samples. They identified 11 differentially expressed protein forms in at least one of the 13 leukemic samples [1]. In the study by Ota et al., the numbers of CD133^+^ stem cells isolated from bone marrow aspirates of healthy individuals were too low for mass spectrometry. However, analyzing cells from a healthy reference group may answer questions of leukemia specificity. Luczak et al. analyzed leukemic cells from 38 patients with AML subtypes M1 and M2 and 17 healthy volunteers using 2D gel electrophoresis and mass spectrometry. In their comparative analysis, they identified 25 differentially regulated proteins, among which they found five potential AML biomarkers. Three proteins (annexin III, L-plastin and 6-phosphogluconate dehydrogenase) were only identified in the AML-M2 group, and the levels of annexin I and actin gamma 1 correlated with resistance to treatment and the time of relapse [2]. In 2013, Bonardi and colleagues paid special attention to the proteins associated with the plasma membrane. They characterized the surface proteome of Fluorescence-Activated Cell Sorter (FACS)—sorted CD34^+^ and CD34^−^ cells from two patients with AML [3]. In CD34^+^ cells, they identified 619 (patient 1) and 386 (patient 2) plasma membrane proteins, containing several previously described and novel proteins. Other studies applying mass spectrometry to analyze the proteome of leukemic (stem) cells focused on AML cell lines [4,5,6]. Aasebo et al. recently reviewed reports in detail on mass spectrometry-based proteomic and phosphosproteomic analyses of AML cell lines and primary AML cells [7]. Luczak et al. provided detailed information on comparative proteomic analyses of AML subtypes in their review [8], while both emphasize the need for more comprehensive proteomic analyses of patient AML samples to identify new leukemia specific biomarkers.

In our study, we analyzed the proteome of CD34^+^CD123^+^ cells from patients with AML. Both CD34 and CD123 are broad markers for AML cells. Several studies claim that Leukemic Stem Cells (LSCs) are present in the AML cell fraction, which co-expresses CD34 and CD123 [9,10,11]. As a reference, we analyzed CD34^+^CD123^+^ normal hematopoietic stem cells from healthy G-CSF mobilized stem cell donors. In addition, CD123 is well-expressed in normal myeloid cells, while expression in CD34^+^ hematopoietic stem and progenitor cells is weaker. A significant co-expression of CD34 and CD123 is still found in common myeloid progenitor cells and in granulocyte/monocyte progenitor cells [12], and there is a low expression of CD123 in a portion of normal hematopoietic stem cells [13]. Since it is proposed that AML stem cells originate from normal hematopoietic stem/progenitor cells (which collect mutations over time) at a stage when CD34 and CD123 were co-expressed [11], healthy CD34^+^CD123^+^ cells appear to be reasonable normal cell counterparts to CD34^+^CD123^+^ AML cells.

Here, we present a method for the identification of novel AML stem cell biomarkers and immunotargets, focusing on membrane-associated and cytosolic proteins from only two million primary CD34^+^CD123^+^ FACS-sorted AML cells. We aim for a more comprehensive analysis of AML precursor cells, compared to previous approaches. By applying a TMT 10-plex labelling-based approach, a direct comparison of the patients suffering from AML with the five healthy donors in one LC-MS becomes feasible, leading to an enhanced coverage of reliably quantifiable proteins. The method described in this pilot study may contribute to the establishment of an attractive diagnostic tool which might be used to validate candidate target molecules expressed by LSCs for personalized therapeutic strategies.

## 2. Materials and Methods 

### 2.1. Patients with AML and Healthy Donors

Blood or bone marrow from patients with AML was collected after written informed consent was obtained and in accordance with the Declaration of Helsinki. The study was approved by the institutional review board of the University Hospital of Dresden (EK-295082014). Patient characteristics are given in Table 1. Leukapheresis products were used from healthy donors mobilized with G-CSF for hematopoietic stem cell transplantation, who gave their written informed consent for scientific use of unused material from the transplant. A leukapheresis product was only utilized for the study when it could no longer be used for clinical purposes (e.g., when the intended recipient had passed away).

### 2.2. Sample Preparation

Mononuclear cells from peripheral blood or bone marrow from patients with AML were isolated by density gradient centrifugation (Biocoll, Biochrom GmbH, Berlin, Germany), and frozen in liquid nitrogen until further use. Leukapheresis products were stored in liquid nitrogen.

### 2.3. Fluorescence Activated Cell Sorting (FACS)

Cells from patients with AML were thawed and stained for FACS-sorting. Healthy CD34^+^ hematopoietic stem cells of leukapheresis products were immunomagnetically enriched (Miltenyi Biotec, Bergisch Gladbach, Germany) before FACS-sorting. Staining, FACS-sorting, and flow-cytometric (re)analyses were performed in accordance with standard protocols. The following fluorochrome-labeled monoclonal antibodies were used to perform cell surface staining: anti-CD34 allophycocyanin (APC-H7) (Miltenyi Biotec, Bergisch Gladbach, Germany), anti-CD123 phycoerythrin-cyanin 7 (PE-Cy7) (BioLegend, San Diego, CA, USA), anti-CD45 BD Horizon V500 (V500) (BD Biosciences, San Jose, CA, USA). For discrimination between live and dead cells, 7-Aminoactinomycin D (7-AAD) was used from BD Biosciences, San Jose, CA. Approximately 2 × 10^6^ 7-AAD^−^ single cells co-expressing CD34, CD45, and CD123 were sorted with a FACSAria II using BD FACSDiva software (Version 8.0.2, BD Biosciences, San Jose, CA, USA, 2016). Sorted cells (purity > 90%) were washed with PBS, snap-frozen in liquid nitrogen, and stored at −80°C until further use.

### 2.4. Proteomic Sample Preparation and Mass Spectrometric Analyses

The cellular content was fractionated using the commercially available Mem-PER Plus Membrane Protein Extraction Kit (Thermo Fisher, Waltham, MA, USA). This was performed with only minor variations to the protocol provided. Briefly, 0.5 mL of cell permeabilization buffer was added, and cells were lysed by three cycles of snap-freeze/thaw method. Samples were centrifuged for 30 min at 20,000× *g*. The supernatant was collected as cytosolic fraction. A 0.2 mL amount of membrane solubilization buffer was added, and samples were incubated for 30 min at 4 °C. Samples were centrifuged for 15 min at 20,000× *g*, and the supernatant was collected as membrane fraction. The cytosolic and membrane fractions were further processed separately. A 10 µg amount of each sample was used for protein digestion (trypsin) and TMT 10-plex labelling as described in the User Manual. Briefly, the peptides obtained from each donor were chemically modified with a specific label, allowing an unambiguous assignment of the corresponding reporter ion in the MS2 scan. Further, the samples of all 10 donors (five AML patients, five healthy donors) were mixed, resulting in two samples corresponding to the cytosolic and membrane fraction, respectively. Samples were evaporated, reconstituted in 0.1% formic acid (FA), and stored at −20 °C until further measurements.

The LC-MS/MS measurements were conducted using a Dionex UltiMate 3000 RSLCnano system coupled to a Q Exactive HF (both Thermo Fisher) as described previously, with only minor changes to the conduct of the process [14]. The peptides were injected at starting conditions of 96% eluent A (0.1% FA in water) and 4% eluent B (0.1% FA in 80% ACN). They were desalinated at steady conditions and a flow of 5 µL/min on a Acclaim PepMap 100 C18 75 µm × 2 cm for 3 min. Peptides were separated by reversed-phase chromatography on a Acclaim PepMap 100 C18 75 µm × 25 cm (both columns from Thermo Fisher) using a 90 min linear increasing gradient from 4% to 25% of eluent B followed by a 25 min linear increase to 50% eluent B. Afterward, the column was flushed up to 99% B and reconstituted to starting conditions of 4% eluent B. Eluting peptides were electrosprayed into the Q Exactive HF at a constant voltage of 1.7 kV using a chip-based electrospray device (TriVersa NanoMate ion source, Advion, Ithaca, NY, USA). The MS1 scans were acquired at R = 120 k, with an AGC target of 3 × 10^6^ and a max. injection time (IT) of 200 ms at a range of 350–1550 *m*/*z*. The top 10 most abundant peptides exceeding a threshold of 5 × 10^3^ were triggered for MS2 acquisition with an isolation window of 1.4 *m*/*z*. Peptides were fragmented at NCE = 38 and the fragment spectra were acquired at R = 60 k, AGC target = 5 × 10^5^ with a max. IT of 200 ms. A fixed first mass was set to 115 *m/z*. All peptides were selected for a dynamic exclusion of 30 s.

### 2.5. Evaluation of Mass Spectrometric Data and Statistics

The acquired MS data were analyzed using the Proteome Discoverer software (Version 2.1, Thermo Fisher). All MS2 spectra were searched against a human reference proteome provided by UniProt Knowledgebase (UniProtKB, retrieved 23 February 2017, 70,714 entries) in target/decoy mode. Both Sequest HT and MS Amanda were used in parallel using the same parameters. Precursor mass tolerance was set to 10 ppm, whereas the fragment mass tolerance was set to 0.05 Da. Two missed cleavages were allowed setting trypsin in ‘full’ digestion mode. Carbamidomethylation of cysteine and TMT 6 plex labels of lysine were set as fixed modifications. Oxidation of methionine and TMT 6 plex labels of peptide N-termini were set as variable modifications. Peptides were evaluated by Percolator. Protein identification was based on at least one unique peptide. The False Discovery Rate (FDR) for peptides and proteins was set to be <0.05. The mass spectrometry proteomics data have been deposited to the ProteomeXchange Consortium via the PRIDE partner repository [15] with the dataset identifier PXD008378. Quantitative values were gained from the TMT reporter ion intensities. Protein with quantitative values from at least two peptides in four of five replicates in both AML and control patients were assigned as quantified. The reporter ion intensities corresponding to the initial samples were median normalized, log2-transformed, and the mean log2 fold-change of AML/control was calculated. Proteins in significantly altered abundance were assigned from Student’s *t*-test (unpaired, two-sided) applying a permutation-based FDR adjustment (s_0_ = 1, 250 permutations). Enrichment analysis of biological processes among significantly altered abundant proteins was conducted by DAVID [16] using only terms limited to an EASE score < 0.05. Regulation of metabolic and signaling pathways as well as upstream regulators was predicted by Ingenuity Pathway Analysis (IPA) [17] using data from bone marrow cells, granulocytes, monocytes, megakaryocytes, and leukemia cell lines. Only pathways with a *p*-value < 0.01 and an assigned z-score were used for further analyses. 

## 3. Results

### 3.1. FACS-Sorting of CD34^+^CD123^+^ Cells

**Figure 1:**
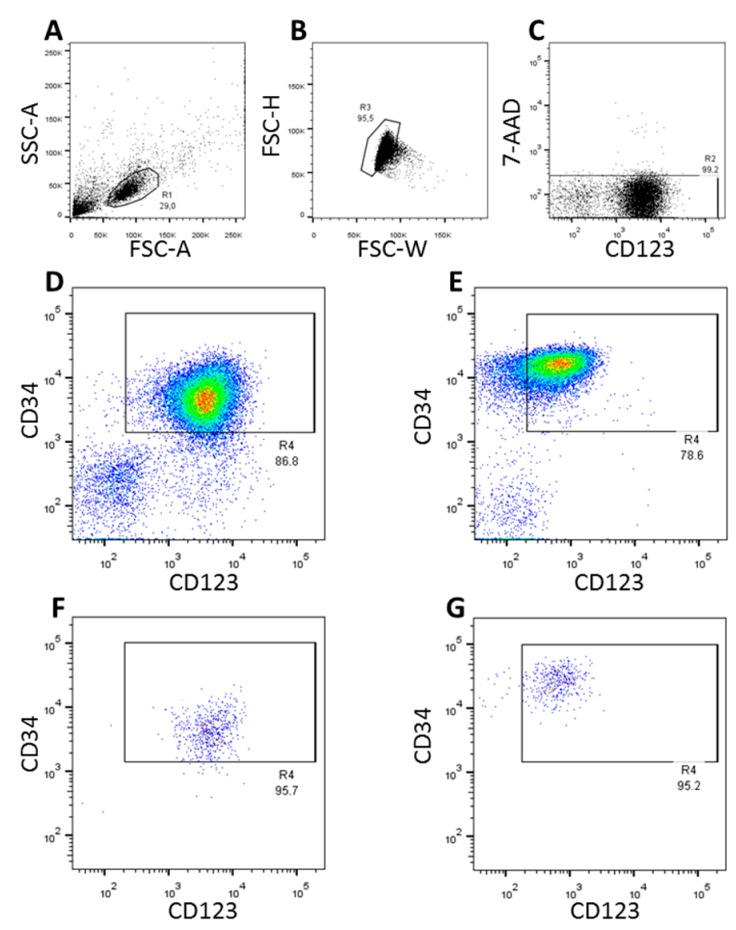
FACS-sort of CD34^+^CD123^+^ cells. Panels (**A**–**E**) depict the gating strategy whereby doublets (**B**) and 7-AAD^+^ (dead) cells (**C**) are excluded. Density plots exemplarily show the expression of CD34 and CD123 in AML cells (**D**) and immunomagnetically isolated CD34^+^ normal hematopoietic stem cells (**E**). Re-analysis of surface expression of CD34 and CD123 in FACS-sorted AML cells and normal hematopoietic cells are shown in Panels (**F**,**G**), respectively.

The CD34 expression tended to be higher in immune-magnetically enriched stem cells from healthy G-CSF mobilized stem cell donors as compared to leukemic cells from patients with AML. Almost all CD34^+^ leukemic cells showed CD123 expression whereas only a part of the normal CD34^+^ cells were also positive for CD123 (Figure 1D,E). In the subsequent FACS-sort, two million CD45^+^7-AAD^−^ hematopoietic stem cells co-expressing CD34 and CD123 were FACS-sorted from each of five patients with AML and from five healthy stem cell donors.

### 3.2. Identification of Proteins in Significantly Altered Abundance from Proteomic Data 

**Figure 2:**
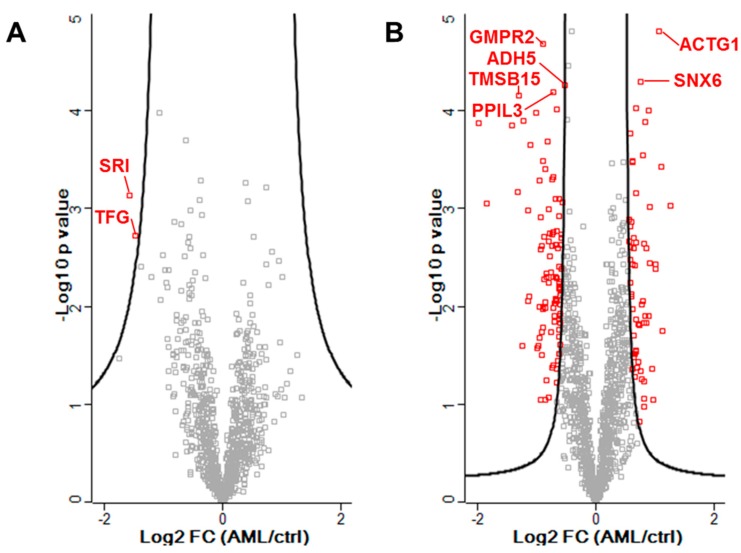
Volcano plot of all reliably quantified proteins in the membrane fraction (**A**) and cytosolic fraction (**B**). In red: proteins with a significantly altered abundance. The solid line indicates the cut-off based on a permutation-based *t*-test (s_0_ = 1, 250 permutations). Marked proteins are further discussed.

By applying a TMT-labeling-based approach, five donors each in healthy condition and suffering from AML could be analyzed in one LC-MS run per cellular fraction. Thus, an enhanced coverage of reliably quantified proteins in combination with even less MS run time was possible. A combined analysis of both fractions allowed a total identification of 2070 proteins in one or both fractions (Supplemental Table S1). In the cytosolic fraction, 1300 proteins were identified in at least four of five replicates of both sample groups, which were quantifiable through having detectable reporter ions originating from at least two peptides. In comparison, 817 proteins were quantified in the membrane fraction. A total of 724 proteins were quantifiable in both cellular fractions. While CD123 was not detected in the proteomic analysis, CD34 showed only a non-significant lower abundance in AML patients compared to healthy donors (fold change = −0.12, *p* = 0.83). Only two proteins of the membrane fraction, Sorcin (SRI) and Protein TFG (TFG), were assigned to be in a significantly altered abundance (assigned from permutation-based FDR adjustment, Figure 2A, Supplemental Table S2). Both SRI and TFG were detected in lower abundance in CD34^+^CD123^+^ AML cells compared to normal HSCs. In the cytosolic fraction 171 proteins (64 up and 107 down) were found to be differentially abundant, also including the aforementioned SRI and TFG in similar alteration as in the membrane fraction (Figure 2B, Supplemental Table S2).

In total, eight proteins were chosen for further consideration. Two proteins (SRI and TFG) were selected after being identified in the membrane fraction in significantly different abundancies. In addition, the six top-ranking proteins from the cytosolic fraction were selected, based on their lowest *p*-value (Table 2). Five of the eight proteins are involved in protein modification processes (ADH5, PPIL3, SNX6), cytoskeleton reorganization (ACTG1, TMSB15A), transmembrane receptor tyrosine kinase activity (ACTG1, SNX6) and/or regulation of the immune response (ACTG1, SNX6). The eight proteins are all cytosolic proteins (GO:0005829/GO:0005737), except for PPIL3, which is located in the nucleus (catalytic step 2 spliceosome, GO:0071013). Some proteins are also present in other cellular components: ACTG1 is embedded in or attached to the plasma membrane, too (inner side of the membrane, GO:0005886), and SNX6 is also an extrinsic component of the membrane (GO:0019898) and present in the nucleus (GO:0005634) according to UniProtKB.

### 3.3. Enrichment Analysis of Significantly Altered Proteins

The proteomic analysis revealed more than 2000 proteins in CD34^+^CD123^+^ AML cells, with 171 proteins in significantly altered abundance in comparison with control HSCs. Thus, analyses beyond the single protein level become feasible. To gain insights into more global changes in CD34^+^CD123^+^ AML cells, an enrichment analysis and clustering of biological processes based on gene ontology (GO) assignments was applied. Fifteen clusters with an enrichment score >2 were assigned (Supplemental Table S3), of which eight were selected for further discussion (Table 3). Thus, AML cells seemed to be affected in multiple processes including metabolic processes concerning isoprenoid and organic hydroxy compounds, response to metal ions, migration and hemostasis, and in cytokine production and signaling via tyrosine kinases. Interestingly, most clusters showed an excess of proteins in lower abundance excluding the transmembrane receptor protein tyrosine kinase and actin filament polymerization cluster. 

**Figure 3:**
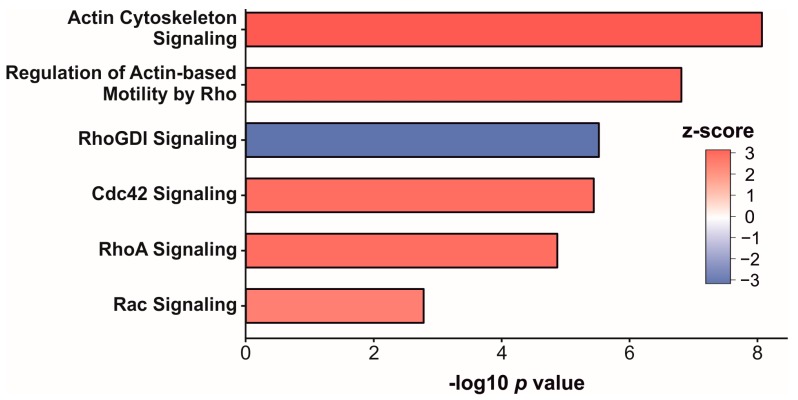
Selection of significantly affected pathways (*p*-value < 0.01) in AML cells compared to healthy donors. Column fill colors indicate the z-score as a measure of the direction of regulation.

To focus on the regulation of signaling pathways, and to predict what upstream regulators might be affected, Ingenuity Pathway Analysis (IPA) was applied. Twenty affected pathways were revealed (*p*-value < 0.01, assigned z-score, Supplemental Table S4). Most of those were involved in signaling regarding the actin polymerization and migration, or regulated Rho GTPase family proteins including Cdc42 signaling (Figure 3). Of all enriched pathways (z-score = 2.4–3.2), RhoGDI signaling was the only one which was downregulated (z-score = −3.0). More interestingly, an upstream regulator analysis predicted the transcription factor SPI1 (z-score = 2.0) to be activated in AML cells. 

## 4. Discussion

Acute myeloid leukemia stem cells (defined by their capacity to initiate and maintain leukemic cell growth) are the most important target cells in immunotherapeutic approaches aimed at the eradication of AML cells. Limited data is available on the proteome of those cells, which is needed for the identification of novel target proteins. Most likely, AML stem cells are part of the CD34^+^CD123^+^ cell fraction of AML blasts [9,10]. Investigating this subset may only represent an approximation for the LSC subset which may vary in each case. In early normal hematopoietic (CD34^+^CD38^−^) stem cells, CD123 expression is low or even absent [13,18,19,20]. In later stages of hematopoiesis CD34 and CD123 are co-expressed in myeloid progenitor cells (but not erythroid precursors), which still possess pluripotency [20]. 

Although the expression levels of CD34 and CD123 tends to differ between normal stem/progenitor cells of healthy donors and AML patients (as depicted in the FACS data presented in Figure 1), CD34^+^CD123^+^ hematopoietic stem/progenitor cells can still be seen as counterparts to leukemic CD34^+^CD123^+^ cells. 

In both cell populations, proliferation and survival are promoted upon binding of interleukin 3 (IL-3) to the CD123 containing IL-3 receptor [21,22]. Due to the broad expression in almost all AML specimens, CD123 itself is already being exploited as a target structure in chimeric antigen receptor (CAR) engineered T-cell and CD123xCD3 bispecific antibody-based therapies [23]. However the risk of marrow ablation might limit the application of anti-myeloid leukemia CAR cells to pre-transplant conditioning where healthy stem cells can be infused after ablation of the CAR cells [24]. In this proof-of-principle-study, we combined cellular fractionation using a commercially available kit with a TMT-labelling approach making a comprehensive comparison of 10 samples available. Applying this method, we were able to identify 171 (cytosolic) and two (membrane) proteins in differential abundance. However, the protocol used for the chemical labelling of the peptides necessitates sample preparation in aqueous solutions, which could entail a bias to miss hydrophobic peptides and proteins. This could explain the gap between the numbers of differentially abundant proteins identified between both fractions and the absence of the CD123 transmembrane protein in the proteomic data set. 

An alternative method aimed at the identification of membrane proteins is the cell surface protein biotinylation and subsequent purification of biotinylated proteins. Membrane proteins with extracellular lysine residues are enriched and are more likely to be seen in mass spectrometric analyses, but higher cell numbers are needed [4,25]. This method might be of special interest for investigating the cell surfaceome of AML stem cells since many immunotherapeutic approaches (e.g., CAR T cells and bi-specific antibodies) target cell surface molecules. Other commercially available or in-house developed procedures to fractionate cellular compartments for LC-MS analyses have been tested by Rockstroh et al. [26] and Hernandez-Valladares et al. [27], using cells of the myeloid lineage and AML cells in particular. Those protocols could be used to focus more specifically on membrane proteins in future experiments or follow up studies. Intracellular proteins that are differentially expressed in AML cells are of potential interest for the development of smart drugs aimed toward blocking vital processes in the leukemic stem cell. Further, immunogenic peptides from over-expressed proteins are more likely to be presented by HLA-molecules and could thus serve as targets for immunotherapy. Leukemia associated protein antigens are, for example, growth factors, mutated proteins, or tumor suppressor inhibitor proteins. The proof of principle for their therapeutic use has already been shown [28]. Clinical trials employing T-cells with genetically engineered T-cell receptors specific for intracellular leukemia antigens are ongoing (e.g., NCT02770820, NCT03326921). For the development of peptide-targeting immunotherapies, mass spectrometric approaches suitable for analyzing the entirety of naturally presented HLA ligands, the HLA ligandome, (as performed by Berlin et al. [29] and others) will become crucial. In our study we found an altered metabolism in AML cells compared to their normal HSC counterparts. One of the most significantly altered proteins was GMPR2, which was involved in the purine-containing compound salvage. Zhang et al. found that overexpressing GMPR2 promoted the monocytic differentiation of cells of the leukemic cell line HL-60 [30]. An affected metabolism could also be confirmed on a more global scale using an enrichment analysis. An enrichment of proteins involved in secondary metabolic processes was found, and organic hydroxyl compounds could be detected among proteins in differential abundance in AML cells. Both clusters comprise an excess of proteins in lower abundance and may point to a dysregulation of these processes.

The most significant upregulated proteins in the cytosolic fractions were Actin, cytoplasmic 2 (ACTG1, 2.11-fold upregulated) and Sorting nexin-6 (SNX6, 1.71-fold upregulated), which might not only be present in the cytoplasm, but also in various other cellular components: ACTG1 is for example also embedded in or attached to the plasma membrane (inner side, GO:0005886), while SNX6 is also an extrinsic component of the membrane (GO:0019898) and present in the nucleus (GO:0005634) according to their annotated gene ontology terms. Another interesting protein that we identified in the proteome of leukemic cells was SNRNP200, although not significantly up-regulated compared to healthy hematopoietic progenitor cells. Only recently, Gillissen et al. found donor-derived antibodies against SNRNP200 in patients with AML after hematopoietic stem cell transplantation. Those antibodies provide anti-leukemic activity after binding to SNRNP200, which is expressed on the cell surface of AML blasts, but which is an intracellular component of the splicosome in normal cells [31]. We also identified cell surface proteins which are accessible to antibodies such as CD44 and CD99, but sample size was too small to detect significant alterations in protein expression. In immunotherapeutic approaches, CD44 has been exploited [32] and CD99 has been proposed as a therapeutic target [33]. The exclusive cell surface expression or significant overexpression of potential target proteins is a prerequisite for antibody or CAR T-cell based immunotherapeutic approaches.

Furthermore, we found the cytokine production to be affected in the AML cells. It was previously shown that AML patients exhibit increased serum levels of IL-13, which may contribute to the inhibition of the cytokine production of those cells [34,35]. Moreover, the regulation of four different signaling pathways was predicted, including or affecting members of the Rho GTPases family (Cdc42, Rac, RhoA, RhoGDI). Rho GTPase family members regulate many cellular processes such as actin cytoskeleton re-organization, adhesion, motility as well as cell proliferation, differentiation and gene expression. An exclusive expression in the hematopoietic lineage was described for Rac2 and RhoH. A lower expression level of RhoH has been found in AML [36]. Together with Rac1, RhoH has an effect in the regulation of the cytoskeleton in hematopoietic cells [37]. In our proteome analyses, we also found a regulation of the actin cytoskeleton signaling pathway. Even the house-keeping cytoplasmic actin 1 (ACTB), a widely used reference protein for quantitative expression analyses, was found to be up-regulated in AML. In their review article, Guo et al. described the up-regulation of ACTB in many solid tumors as well as in leukemia. They concluded that ACTB might be involved in the pathogenesis of certain cancers; that ACTB might not be a valid reference protein for quantifying expression levels; and that ACTB might be a potential biomarker of cancer [38]. The signaling pathway analysis also predicted a potential activation of the transcription Factor PU.1 (SPI1).The transcription factor SPI1 is a key regulator of various steps of hematopoiesis including HSC self-renewal and myeloid differentiation. If dysregulated, SPI1 can function as a tumor suppressor or as oncogene [39]. Thus, we show the potential of comprehensive proteome analyses to contribute to a better understanding of AML leukemogenesis.

In summary, we identified 2070 proteins in samples of only 2 × 10^6^ cells, fractionated into a cytosolic and a membrane fraction. To validate good candidate proteins for immunotherapies, mass spectrometrical analyses would be necessary for a larger cohort of patients with a defined AML subtype as well as validation experiments using flow-cytometry and/or Western Blot techniques. Our method also allows analyses for individual patients using no more than 10 ml peripheral blood at a time point when CD34^+^CD123^+^ AML cells are present in detectable levels in blood (~4 GPt/L). This provides a rationale to further investigate options of combining proteomic and genomic analyses of AML cells, so called proteogenomic approaches, for an identification of individualized AML biomarkers as proposed by others [40].

## Figures and Tables

**Table 1:** Patient and donor characteristics.

Patient/Donor ID	Sex	Age	Disease Characteristics/Donor Information
AML_033	f	61	AML FAB M4, Complex aberrant karyotype, negative for *FLT3*-ITD, *NPM1*, *CEBPA*
AML_036	m	66	sAML from CMML, trisomy 8, deletion 21q, duplication 15q11/15q26, negative for *FLT3*-ITD, *NPM1*, *CEBPA*
AML_037	f	60	AML FAB M5, normal karyotype, *FLT3*-ITD^+^, *NPM1* mutation type D
AML_044	m	71	AML FAB M1, normal karyotype, negative for *FLT3*-ITD, *NPM1*, *CEBPA*
AML_049	f	18	AML FAB M1, normal karyotype, *FLT3*-ITD^low^, negative for *NPM1, CEBPA*
DON_004	m	39	all donors passed a medical work-up and were released for stem cell donation; the following conditions were excluded: active infections such as viral hepatitis, AIDS, syphilis, malaria; history of malignant tumors or systemic autoimmune diseases; medically significant abnormalities of the differential blood count
DON_005	f	25	
DON_011	f	40	
DON_012	m	44	
DON_013	m	20	

**Abbreviations:** FAB, French American British Classification; wt, wild type; *FLT3*-ITD, fms-like tyrosine kinase 3-internal tandem duplication; *NPM1*, Nucleophosmin 1; sAML, secondary acute myeloid leukemia; CMML, chronic myelomonocytic leukemia; *CEBPA*, CCAAT/enhancer-binding protein alpha; *FLT3*-ITDlow, ratio of the mutant to wild type allele <0.5.

**Table 2:** Proteins with differentially altered abundance.

Cytosolic/Membrane Fraction	Accession ID	Gene ID	Description	Number of Peptides	LOG_2_ [FC (AML/DON)]	*p*-Value
CF	P63261	ACTG1	Actin, cytoplasmic 2	67	1.08	<0.0001
CF	Q9P2T1	GMPR2	GMP reductase 2	3	−0.90	<0.0001
CF	Q9UNH7	SNX6	Sorting nexin-6	3	0.77	<0.0001
CF	P11766	ADH5	Alcohol dehydrogenase class-3	2	−0.53	<0.0001
CF	Q9H2H8	PPIL3	Peptidyl-prolyl cis-trans isomerase-like 3	3	−0.72	<0.0001
CF	P0CG34	TMSB15A	Thymosin beta-15A	2	−1.31	<0.0001
MF	P30626	SRI	Sorcin	4	−1.57	0.0007
MF	Q92734	TFG	Protein TFG	3	−1.49	0.0019

**Abbreviations:** CF, cytosolic fraction; MF, membrane fraction; FC, fold-change; Accession ID, Protein identifier according to UniProtKB; Gene ID, Gene name according to NCBI.

**Table 3:** Results of a gene ontology (GO)-term (biological process) enrichment analysis including selected clusters.

Cluster	Enrichment Score	Number of Proteins Sign. Higher Abundance	Number of Proteins Sign. Lower Abundance
cellular response to endogenous stimulus (GO:0071495)	3.1	25	35
cellular response to metal ion (GO:0071248)	2.7	9	22
isoprenoid metabolic process (GO:0006720)	2.6	2	14
tissue homeostasis (GO:0001894)	2.6	6	7
negative regulation of cytokine production (GO:0001818)	2.5	6	15
organic hydroxy compound metabolic process (GO:1901615)	2.3	3	20
transmembrane receptor protein tyrosine kinase signaling pathway (GO:0007169)	2.3	20	16
actin filament polymerization (GO:0030041)	2.2	25	16

## Supplementary Materials

The following are available online at http://www.mdpi.com/2227-7382/6/1/11/s1, Table S1: Complete list of mass spectrometrical identified proteins, Table S2: List of differentially abundant proteins, Table S3: Results from functional enrichment and clustering, Table S4: List of affected pathways based on Ingenuity Pathway Analysis (IPA).
